# Seasonal and anthropogenic influences on bacterioplankton communities: ecological impacts in the coastal waters of Qinhuangdao, Northern China

**DOI:** 10.3389/fmicb.2024.1431548

**Published:** 2024-06-19

**Authors:** Qiuzhen Wang, Jia Yu, Xiaofang Li, Yong Zhang, Jianle Zhang, Jianyan Wang, Jiandong Mu, Xinping Yu, Ruixue Hui

**Affiliations:** ^1^Ocean College, Hebei Agricultural University, Qinhuangdao, China; ^2^Hebei Key Laboratory of Nutrition Regulation and Disease Control for Aquaculture, Qinhuangdao, China; ^3^Department of Ocean Survey, Qinhuangdao Marine Center of the Ministry of Natural Resources, Qinhuangdao, China; ^4^Department of Life Sciences, National Natural History Museum of China, Beijing, China; ^5^Ecological Environment Research Department, Hebei Ocean and Fisheries Science Research Institute, Qinhuangdao, China

**Keywords:** bacterioplankton community, seasonal changes, human activities, coastal waters, ecological functions, nutrient cycles

## Abstract

Marine bacterioplankton play a crucial role in the cycling of carbon, nitrogen, and phosphorus in coastal waters. And the impact of environmental factors on bacterial community structure and ecological functions is a dynamic ongoing process. To systematically assess the relationship between environmental changes and bacterioplankton communities, this study delved into the spatiotemporal distribution and predicted metabolic characteristics of bacterioplankton communities at two estuarine beaches in Northern China. Coastal water samples were collected regularly in spring, summer, and autumn, and were analyzed in combination with environmental parameters and bacterioplankton community. Results indicated significant seasonal variations in bacterioplankton communities as Bacteroidetes and Actinobacteria were enriched in spring, Cyanobacteria proliferated in summer. While Pseudomonadota and microorganisms associated with organic matter decomposition prevailed in autumn, closely linked to seasonal variation of temperature, light and nutrients such as nitrogen and phosphorus. Particularly in summer, increased tourism activities and riverine inputs significantly raised nutrient levels, promoting the proliferation of specific photosynthetic microorganisms, potentially linked to the occurrence of phytoplankton blooms. Spearman correlation analysis further revealed significant correlations between bacterioplankton communities and environmental factors such as salinity, chlorophyll *a*, and total dissolved phosphorus (TDP). Additionally, the metabolic features of the spring bacterioplankton community were primarily characterized by enhanced activities in the prokaryotic carbon fixation pathways, reflecting rapid adaptation to increased light and temperature, as well as significant contributions to primary productivity. In summer, the bacterial communities were involved in enhanced glycolysis and biosynthetic pathways, reflecting high energy metabolism and responses to increased light and biomass. In autumn, microorganisms adapted to the accelerated decomposition of organic matter and the seasonal changes in environmental conditions through enhanced amino acid metabolism and material cycling pathways. These findings demonstrate that seasonal changes and human activities significantly influence the structure and function of bacterioplankton communities by altering nutrient dynamics and physical environmental conditions. This study provides important scientific insights into the marine biological responses under global change.

## 1 Introduction

The coastal region of Bohai Sea, as one of the fastest urbanizing and developing coastal areas in China, faces significant water quality pollution due to inland pollution input and the hydrogeological conditions of the Bohai Sea. This issue is particularly acute in the western offshore region of Bohai Bay, where the water exchange capacity is extremely poor (Wang et al., 2019). Inorganic nitrogen, active phosphorus and petroleum hydrocarbons are among the key pollutants in seawater (Peng, 2015), posing a serious threat to the Bohai Sea ecosystem (Peng, 2015; Zhou et al., 2021). Nitrogen and phosphorus from inland sources enter the Bohai Sea via rivers and submarine groundwater discharge (SGD), causing severe eutrophication, which adversely impacts water quality and ecosystem stability (Zhou et al., 2021). Moreover, the seasonal increase in inorganic nitrogen and active phosphorus concentrations frequently leads to harmful algal blooms (HABs) in coastal waters, particularly during the summer tourist season. This thereby seriously affects coastal environments and economic activities. Although water quality in the Bohai Sea has shown gradual improvement since 2012, recent conditions remain concerning (Zhou et al., 2021).

As key participants in nearshore biogeochemical cycles, microorganisms play a crucial role in maintaining the balance of marine ecosystems and degrading high-molecular hydrocarbons and nitrogen- or sulfur-containing organic compounds (Tang et al., 2022; Wang et al., 2022; Glibert et al., 2023; Yu et al., 2023). Microbial diversity, community composition and distribution are significantly influenced by their natural habitat. During HABs induced by coastal eutrophication, microorganisms establish close interactions with microalgae to adapt to the surrounding environment (Worden et al., 2015; Li et al., 2020; Sidhu et al., 2023). Certain functional microbial communities become enriched during specific bloom stages. For instance, the quantity and composition of dissolved polysaccharides during phytoplankton blooms significantly impact the composition of planktonic bacterial communities and enriched the branch with polysaccharide degradation function (Sidhu et al., 2023). These microorganisms can serve as bioindicators for monitoring the progression of algal blooms (Shao et al., 2020; Shi et al., 2023).

As transitional zones between land and sea, estuarine ecosystems exhibit complex biogeochemical processes, with nitrogen cycling playing a central role (Chen et al., 2021; Wei et al., 2023). Different types of estuaries, subjected to varying degrees of anthropogenic influence, show significant differences in their pollutant composition and functional microbial community structure (Freeman et al., 2019; Li et al., 2019; Jeamsripong et al., 2020; Grogan and Mallin, 2021; Wang C. et al., 2021). For instance, urbanized estuaries often face significant pollution from heavy metals, organic contaminants and nutrients, primarily due to residential, industrial and port activities (Peng, 2015; Chen et al., 2019; Wang et al., 2022). These pollutants affect water quality and can also indirectly impact the entire ecosystem by altering the structure and function of microbial communities. In urban estuaries, Desulfobacterales and its subfamily Desulfobulbaceae, as well as Rhodobacterales and its subfamily Rhodobacteraceae, associated with nitrogen and phosphorus cycling, are easily inhibited by heavy metals (Yi et al., 2021). Zinc oxide nanoparticles are somewhat toxic to bacterial communities in estuaries (Feng et al., 2020), while copper nanoparticles at certain concentrations can promote the activity of denitrifying bacterial communities (Costa et al., 2021). Therefore, the impact of estuarine pollutants on microbial communities remains uncertain, necessitating comprehensive analyses of environmental factors across various estuarine types to uncover their specific driving mechanisms. Furthermore, most studies indicate that salinity, temperature and nutrients are key indicators affecting bacterial community in estuaries (Santos et al., 2020; Wu et al., 2022). However, the overall influence of different forms of carbon, nitrogen, and phosphorus on estuarine bacterial communities remains largely unknown.

In this article, two important tourist beaches in Qinhuangdao, namely, West Beach and Dongshan Beach, were studied in terms of the bacterioplankton communities, ecological function and environmental parameters in spring, summer, and autumn. The purpose of this article is to reveal the spatial and temporal distribution, ecological functions, and main environmental factors of the bacterioplankton communities under the influences of human activities in the innermost area of the semi-enclosed Bohai Bay. This study will provide an important scientific basis for the formulation of management and protection policies for coastal estuary beaches.

## 2 Materials and methods

### 2.1 Study area

**FIGURE 1:**
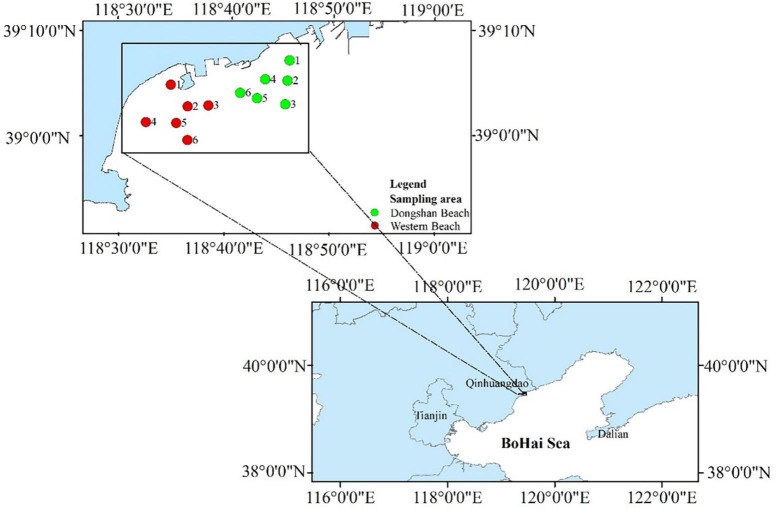
Sampling stations in the present study.

The sampling area was located at two coastal beaches (West Beach and Dongshan Beach) in Qinhuangdao City, Hebei Province of Northern China (Figure 1). West Beach is located near the mouth of Tanghe River into the Bohai Bay, surrounded by residential areas and leisure resorts. It is one of the important public beaches in Qinhuangdao, and tourists are numerous especially in summer. There are two artificial islands built in this area, one of which has been basically dismantled, but have a great impact on the hydrodynamic conditions and seawater quality in this area. Especially in summer, due to the increase of surface runoff, changes in water temperature, dissolved oxygen, and other environmental parameters, ecological disasters such as phytoplankton blooms and green tide often break out. This seriously affects the ecological environment and makes coastal tourism suffer heavy losses. In addition, Dongshan Beach is close to the West Beach and Qinhuangdao Port. The thermal power plant wastewater carries the residential wastewater into this area through the estuary of the Xinkai River. It is also one of the famous tourist beaches in Qinhuangdao.

### 2.2 Sample collection

A total of 12 sampling stations were established, including six at Western Beach and six at Dongshan Beach (Supplementary Table 1). Seawater samples were collected at a depth of 0.5 m each month during spring (April and May), summer (June, July, and August) and autumn (September and October) of 2021. The seawater samples were collected using pre-sterilized 2 L glass bottles, with two bottles of seawater collected at each station. These samples were tightly wrapped in black plastic bags and ice packs, and transported back to the laboratory as quickly as possible. Sample labels from Western Beach in April, August, and October were prefixed with A, C, and E, while samples from Dongshan Beach were labeled with B, D, and F, respectively, in order to denote station names.

### 2.3 Determination of environmental parameters

Several seawater physical parameters, including depth, temperature, pH, turbidity, and salinity, were measured using a portable YSI Pro Plus multiparameter instrument (YSI Inc., United States). Other basic physicochemical parameters, including DO, NO_2_^–^, NO_3_^–^, NH_4_^+^, PO_4_^3–^, reactive silicate and chlorophyll *a*, were determined according to the “Specification for the Oceanographic Survey” (GB/T 12763.4-2007). Additionally, 500 ml of seawater was collected in polyethylene bottles from each station, and 1.0 ml of 50% sulfuric acid solution was added and mixed. And then the bottle was tightly capped for storage. This method has an effective preservation time of 1 month for determining total nitrogen (TN), total phosphorus (TP), and total organic carbon (TOC). A total of 1,350 ml of seawater was collected from each station and filtered through nine 0.45 μm mixed cellulose ester microporous filters (pre-soaked in 1% hydrochloric acid for 12 h, rinsed with distilled water until neutral, and then stored in distilled water). The filtrate was divided into polyethylene bottles for the determination of total dissolved nitrogen (TDN), total dissolved phosphorus (TDP), and dissolved organic carbon (DOC). The collected filters were used to measure particulate inorganic nitrogen (PIN) and particulate organic phosphorus (POP).

Total organic carbon and DOC were determined using a total organic carbon analyzer, while TN, TP, TDN, and TDP were analyzed following the “Specification for the Oceanographic Survey” (GB/T 12763.4-2007). To analyze PIN and POP, filters were placed in digestion bottles, and 25 ml of 0.1 mol/L hydrochloric acid was added to the bottle and shaken for 2 h. The supernatant was collected for PIN analysis, while the remaining filter was used for POP analysis (Zhou et al., 2019). The supernatant was transferred to a digestion bottle with potassium persulfate solution for digestion, and PIN was determined according to the TN method. The remaining filter was placed in a polyethylene bottle with 25 ml of ultrapure water and 2.5 ml of potassium persulfate. It was then heated for 30 min in an autoclave for digestion. After cooling to room temperature, the POP was determined following the TP method. Furthermore, particulate nitrogen (PN) was resulted from the subtraction of TDN from TN, and particulate organic nitrogen (PON) was resulted from the subtraction of PIN from PN.

### 2.4 DNA extraction and high-throughput sequencing

Seawater samples from April (spring), August (summer), and October (autumn) were selected, and 1 L seawater was filtered onto a 0.22 μm polycarbonate membrane (Millipore Isopore, USA). The filter was collected and total DNA was extracted using the E.N.Z.A.™ Water DNA Kit D5525-01 (Omega, USA) following the manufacturer’s instructions. The DNA samples were sent to Hangzhou Lianchuan Biotechnology Co., Ltd. for high-throughput sequencing. The DNA quantification was performed using a UV spectrophotometer. For PCR amplification, the universal primers 341F (5′-CCTACGGGNGGCWGCAG-3′) and 805R (5′-GACTACHVGGGTATCTAATCC-3′) were used to amplify the bacterial 16S rRNA gene using total DNA as the template. The PCR procedure was as follows: initial denaturation at 98 °C for 30 s, followed by 35 cycles of 98 °C for 10 s, 54 °C for 30 s and 72 °C for 45 s, and a final extension at 72 °C for 10 min. The purified PCR products were evaluated using an Agilent 2100 Bioanalyzer (Agilent, USA) and the Library Quantification Kit from Illumina (Kapa Biosciences, Woburn, MA, USA). Qualified sequencing libraries (with non-repeating Index sequences) were diluted and mixed proportionally based on the desired sequencing output. Paired-end sequencing was conducted using a NovaSeq 6000 SP Reagent Kit (500 cycles) on the NovaSeq 6000 platform for 2 × 250 bp paired-end sequencing. For the paired-end sequencing data, samples were initially separated based on their barcode information, and adapter and barcode sequences were removed.

#### 2.4.1 Data merging and filtering

Each pair of paired-end reads was merged into a longer tag based on the overlap region using FLASH (v1.2.8) with the parameters “-m 10 -M 100 -x 0.25 -t 1 -z.” Quality scanning through a sliding window approach was performed on the sequencing reads, with a default window size of 100 bp. If the average quality within a window dropped below 20, the part of the read from the start of the window to the 3′ end was trimmed (Software: fqtrim, Parameters: “-P 33 -w 100 -q 20 -l 100 -m 5 -p 1 -V -o trim.fastq.gz”). Reads truncated to less than 100 bp, containing more than 5% indeterminate bases (N), or identified as chimeric sequences were discarded. Chimeras were removed using the default settings of Vsearch (v2.3.4).

#### 2.4.2 DADA2 denoising

The DADA2 pipeline was invoked through qiime dada2 denoise-paired for length filtering and denoising. This process generated ASV (feature) sequences and an ASV (feature) abundance table while removing singletons ASVs (i.e., ASVs that appear only once across all samples, by default operation).

### 2.5 Data analysis and statistics

The analysis of alpha and beta diversity was conducted using the ASV feature sequences obtained to assess the species complexity within and between groups. Alpha diversity was analyzed with community richness, Shannon diversity, and Pielou’s evenness, which were calculated using QIIME2.^1^ For comparative analysis of bacterial communities, one-way ANOVA and Duncan’s multiple range test were utilized with significance determined at *P* < 0.05. Beta diversity metrics were also computed using QIIME2, and visualizations were generated using R package (v3.5.2). Species annotation was performed using the SILVA database and NT-16S database based on the ASV sequence file with a confidence threshold of 0.7 for species annotation. The species abundance in each sample was quantified from the ASV counts. Furthermore, functional prediction of the bacterial communities was conducted using PICRUSt2 software. OmicStudio tools^2^ were used for data analysis, including principal component analysis (PCA) for bacterial communities among different samples, Spearman correlation analysis between various environmental parameters, redundancy (RDA) analysis between the bacterial communities and environmental factors, bubble map, correlation heatmap cluster analysis, as well as the above figures creation. Besides, diagrams of the above indicators were implemented using the R package (v3.5.2).

## 3 Result

### 3.1 Bacterioplankton diversity

**FIGURE 2:**
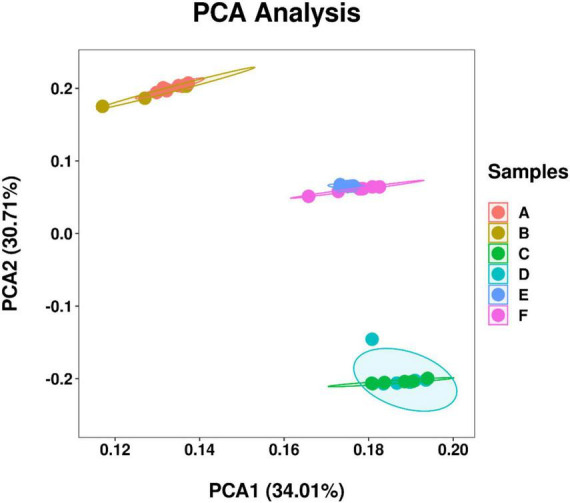
Principal component analysis (PCA) of bacterioplankton communities among samples in different station and sampling seasons in the coastal waters of the Western Beach and Dongshan Beach. Circles in different color represent coastal waters of Western Beach in April (A), August (B), and October (C), as well as Dongshan Beach in April (D), August (E), and October (F).

**FIGURE 3:**
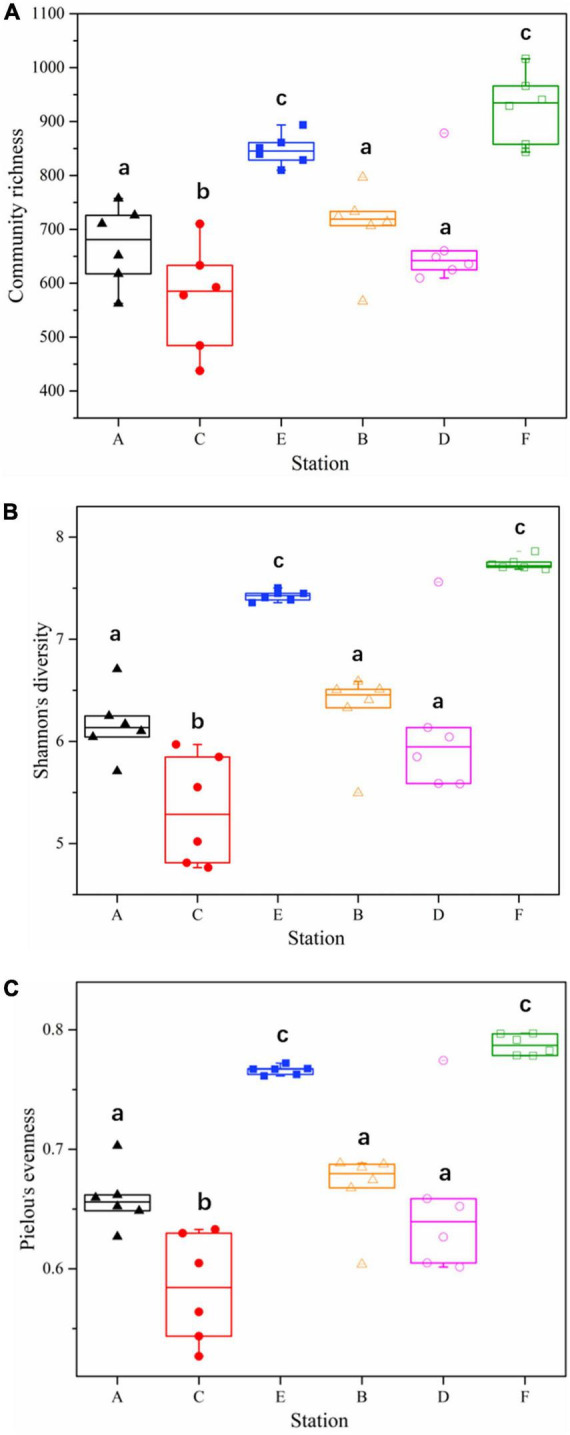
Alpha diversity of bacterioplankton communities among samples in different station and sampling seasons in the coastal waters of the Western Beach and Dongshan Beach. **(A)** Community richness; **(B)** Shannon’s diversity; **(C)** Pielou’s evenness. Capital letters on the horizontal axis represent sampling information (A, Western Beach in spring; B, Dongshan Beach in spring; C, Western Beach in summer; D, Dongshan Beach in summer; E, Western Beach in autumn; F, Dongshan Beach in autumn). Small letters “a–c” represent the results of significance analysis. The same letters indicate no significant difference between stations (*P* > 0.05), while the different letters indicate significant difference between stations (*P* < 0.05).

A total of 26,263 ASV feature sequences were obtained in this study. PCA based on Bray–Curtis distances indicate that season was a key factor influencing the temporal and spatial distribution of bacterioplankton communities at both Western Beach and Dongshan Beach (Figure 2). All samples were grouped from top to bottom into three clusters, namely spring, autumn, and summer. The spring and summer clusters were quite distant from each other, suggesting significant differences in bacterioplankton community composition between these two seasons. Overall, the autumn samples at both beaches exhibited significantly higher community richness, Shannon diversity index, and Pielou’s evenness index compared to the spring and summer samples (*P* < 0.05) (Figure 3). This suggests that species diversity and evenness were both higher in autumn than in spring and summer. Besides, higher community richness, Shannon diversity and Pielou’s evenness were positively correlated with DIN, PO_4_^3–^ and DOC (Supplementary Figure 1). In addition, chlorophyll *a*, PN and PP were primarily enriched in summer, with phytoplankton contributing to PON and POP (Supplementary Figure 1).

### 3.2 Bacterioplankton communities

**FIGURE 4:**
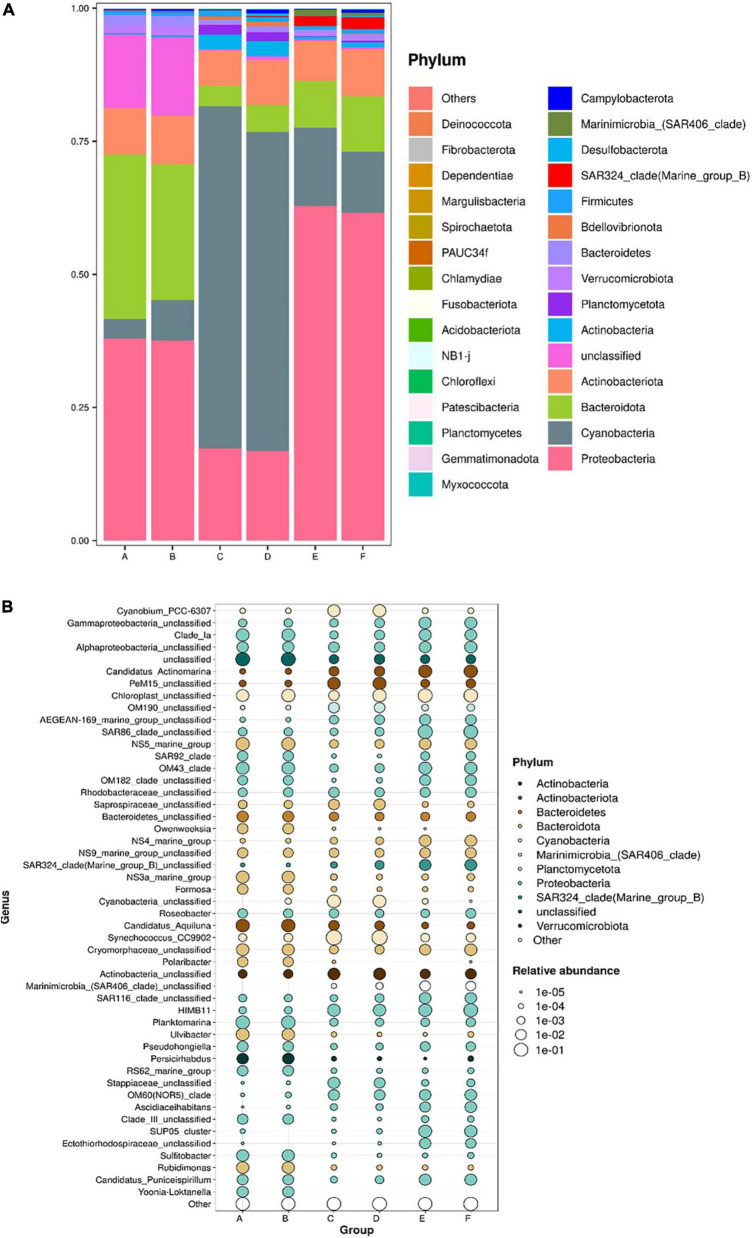
Bacterioplankton communities at the phylum level **(A)** and their composition at the genus level **(B)** in coastal waters of the Western Beach and Dongshan Beach. Capital letters on the horizontal axis represent sampling information (A, Western Beach in spring; B, Dongshan Beach in spring; C, Western Beach in summer; D, Dongshan Beach in summer; E, Western Beach in autumn; F, Dongshan Beach in autumn).

The dominant bacterioplankton phyla at both beaches were Pseudomonadota (formerly Proteobacteria), Cyanobacteria, Bacteroidota (formerly Bacteroidetes), and Actinobacteria (Figure 4). However, the proportions of Pseudomonadota, Cyanobacteria, and Bacteroidota within the total bacterioplankton population varied significantly across spring, summer, and autumn. While Actinobacteria showed relatively stable proportions across varied seasons. Pseudomonadota had the highest proportions in spring and autumn, accounting for up to 60% in autumn. Cyanobacteria were the most abundant in summer, reaching 60% at both beaches. Bacteroidota was significantly enriched in spring, with a notable abundance of unknown species comprising about 20% of the spring bacterial population. Additionally, SAR324, a subgroup within Pseudomonadota, was notably enriched in autumn.

**FIGURE 5:**
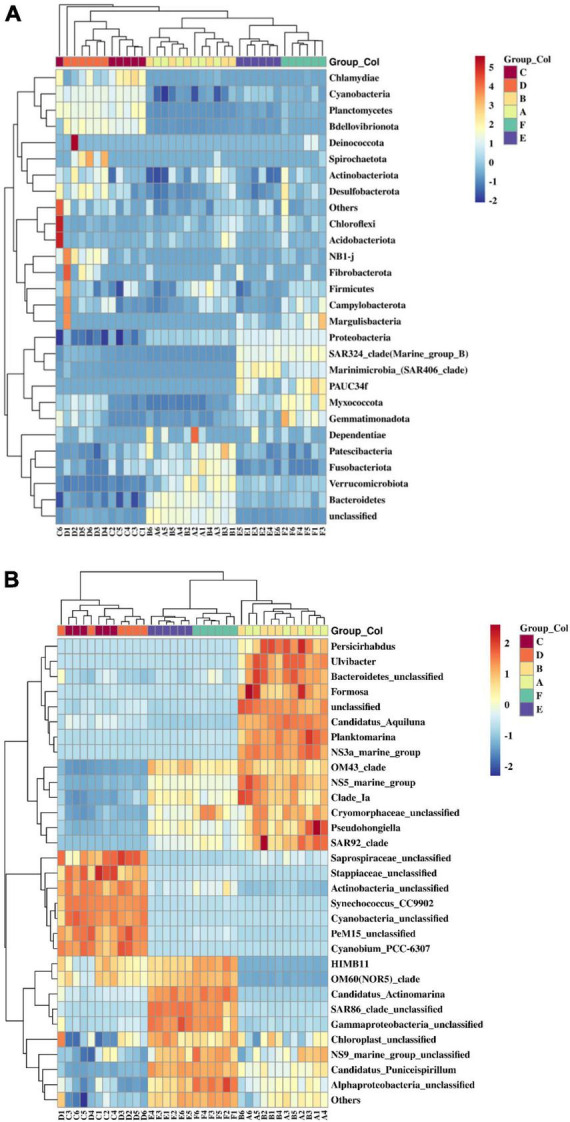
Heatmap of bacterioplankton communities at the phylum level with top 28 **(A)** and genus level with top 31 **(B)** in coastal waters of two beaches of the present study. Capital letters in the sample name mean sampling information (A, Western Beach in spring; B, Dongshan Beach in spring; C, Western Beach in summer; D, Dongshan Beach in summer; E, Western Beach in autumn; F, Dongshan Beach in autumn).

The clustering heatmap indicates that seasonal variations obviously influence the bacterioplankton community in coastal seawater samples in the present study (Figure 5). At the phylum level, Bacteroidetes, Fusobacteriota, Patescibacteria, Verrucomicrobiota, Bacteroidota, and unclassified bacteria clustered in spring samples. Chlamydiae, Cyanobacteria, Bdellovibrionota, and Planctomycetes grouped together in summer samples. While Pseudomonadota, SAR324_clade (Marine_group_B), Marinimicrobia (SAR406 clade), PAUC34f, Myxococcota, and Gemmatimonadota clustered in autumn samples. At the genus level, microbes predominantly enriched in spring samples included *Persicirhabdus*, *Ulvibacter*, Bacteroidetes unclassified, Formosa, unclassified species, *Candidatus Aquiluna*, *Planktomarina*, NS3a marine group, OM43 clade, NS5 marine group, Clade Ia, Cryomorphaceae unclassified, *Pseudohongiella*, and SAR92 clade. In the summer samples, Saprospiraceae unclassified, Stappiaceae unclassified, Actinobacteria unclassified, *Synechococcus* CC9902, *Cyanobacteria* unclassified, PeM15 unclassified, and *Cyanobium* PCC-6307 were enriched. Meanwhile, bacterioplankton communities primarily enriched in autumn samples were HIMB11, OM60 (NOR5)_clade, *Candidatus*, *Actinomarina*, SAR86 clade unclassified, Gammaproteobacteria unclassified, Chloroplast unclassified, NS9_marine_group_unclassified, *Candidatus*, *Puniceispirillum*, and Alphaproteobacteria unclassified. Additionally, the offshore distance also affected bacterioplankton community structure in this study (Supplementary Figure 2). Planctomycetes, Chloroflexi, and Acidobacteriota were significantly enriched at offshore stations in the Western Beach. NB1-j, SAR324 clade, Synergistota, Margulisbacteria, Firmicutes, and Campylobacterota were enriched near river estuaries at inshore stations in Dongshan Beach, where environmental pressure selectively enriched specific bacterioplankton communities.

### 3.3 Influencing factors of bacterioplankton community distribution

**FIGURE 6:**
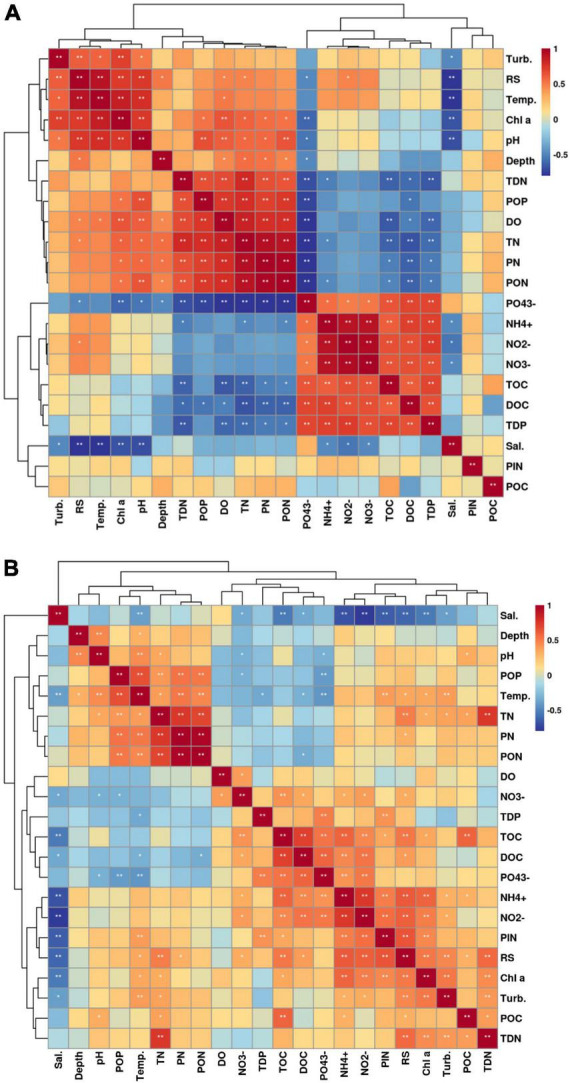
Spearman correlation heatmap of the environment parameters of coastal seawater in the Western Beach **(A)** and Dongshan Beach **(B)**.

We measured the basic environmental parameters of seawater as well as the concentrations of different forms of carbon, nitrogen, and phosphorus at these two beaches. The nutrients concentration exhibited different characteristics of seasonal variation (Supplementary Table 2). The salinity was the highest in spring, followed by the autumn and summer. The concentrations of NO_2_^–^, NO_3_^–^, PO_4_^3–^, TOC, and DOC exhibited the highest values in autumn in both Western Beach and Dongshan Beach. While the concentrations of Chl *a*, TN, TDN, PN, PON, and POP exhibited the highest values in summer. Additionally, the concentrations of PP and PIP were higher in summer than that in autumn. Furthermore, the concentration of NH_4_^+^ exhibited the highest value in autumn, followed by that in summer and spring in Western Beach. In contrast, it showed the highest value in summer, followed by that in autumn and spring in Dongshan Beach. Spearman correlation analysis indicated that salinity at both beaches showed a significant negative correlation with nitrate, nitrite and ammonium ions (*P* < 0.05) (Figure 6). Chlorophyll *a* was significantly positively correlated with PON and POP (*P* < 0.05) and significantly negatively correlated with phosphate (*P* < 0.05). At Dongshan Beach, chlorophyll *a* also exhibited a significant negative correlation with total dissolved phosphorus (TDP) (*P* < 0.05).

**FIGURE 7:**
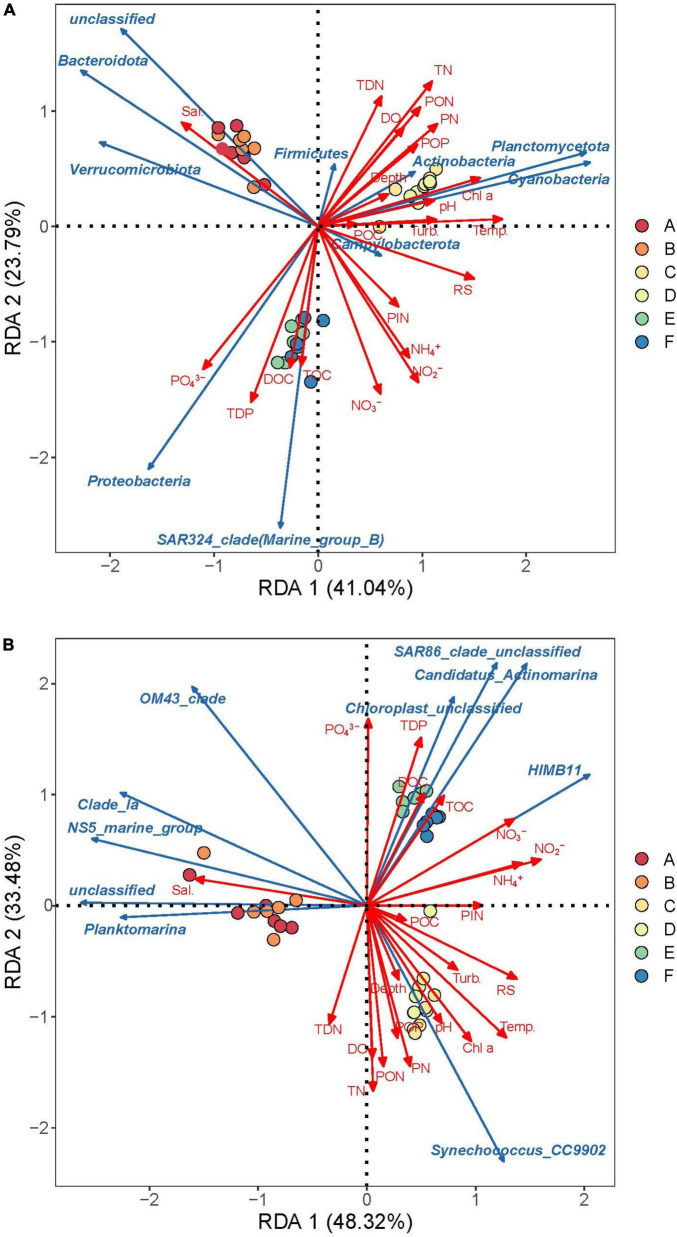
Redundant analysis (RDA) of environmental factors and bacterioplankton communities with top 10 phylum **(A)** and top 10 genera **(B)**. Capital letters with different colored circles represent sampling information (A, Western Beach in spring; B, Dongshan Beach in spring; C, Western Beach in summer; D, Dongshan Beach in summer; E, Western Beach in autumn; F, Dongshan Beach in autumn). Sal., salinity; Turb., turbidity; RS, reactive silicate; Temp., temperature; DO, dissolved oxygen; TOC, total organic carbon; DOC, dissolved organic carbon; POC, particulate organic carbon; TN, total nitrogen; PN, particulate nitrogen; PON, particulate organic nitrogen; TDN, total dissolved nitrogen; PIN, particulate inorganic nitrogen; TDP, total dissolved phosphorus; POP, particulate organic phosphorus.

Correlation analysis between the abundance of dominant bacterioplankton groups and environmental factors revealed that some dominant phyla exhibited strong correlations with specific environmental factors (Figure 7 and Supplementary Figure 3). In spring samples, Bacteroidota, Verrucomicrobiota, and unidentified high-abundance taxa were clearly enriched, showing a significant positive correlation with salinity (*P* < 0.05), and a significant negative correlation with PIN, ammonium, nitrite, and nitrate. Cyanobacteria (mainly *Synechococcus*), Planctomycetota, and Actinobacteriota dominated summer samples, exhibiting significant positive correlations with chlorophyll *a*, POP, PN, and PON (*P* < 0.05). Proteobacteria and SAR324 dominated autumn samples and showed significant positive correlations with TOC, DOC, TDP, and PO_4_^3–^ (*P* < 0.05).

### 3.4 Functional predictions of bacterioplankton communities

**FIGURE 8:**
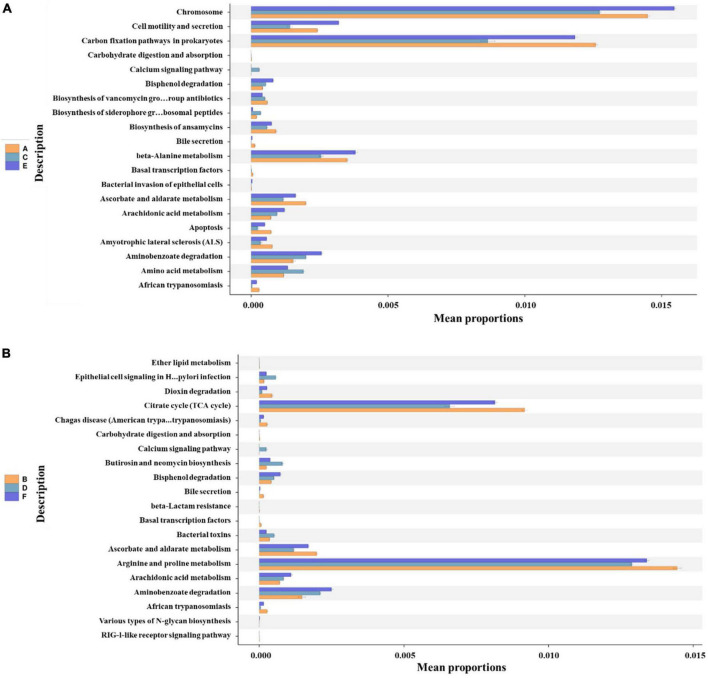
The top 20 functional predictions of bacterioplankton communities with significant differences (*P* < 0.05) at KEGG level 3 in the Western Beach **(A)** and Dongshan Beach **(B)**. Capital letters in the sample name mean sampling information (A, Western Beach in spring; C, Western Beach in summer; E, Western Beach in autumn; B, Dongshan Beach in spring; D, Dongshan Beach in summer; F, Dongshan Beach in autumn).

In the present study, functional predictions of the bacterioplankton communities in coastal waters of Western Beach and Dongshan Beach were conducted using PICRUSt2 at KEGG level 3 (Figure 8 and Supplementary Figure 4). In spring, KEGG pathway analysis revealed obvious enrichment in prokaryotic carbon fixation pathways, indicating an increase in photosynthesis activity due to more sunlight and promoting carbon fixation to enhance primary productivity. While the enhanced carbohydrate metabolism suggested that microbial communities were actively engaged in the breakdown and assimilation of carbon sources. In summer, KEGG pathway analysis revealed enrichment in glycolysis/gluconeogenesis pathways, suggesting active microbial participation in energy production and carbohydrate metabolism. Additionally, the enrichment in lysine and other essential amino acid biosynthesis pathways as well as glutathione metabolism, indicates rapid bacterial (majorly cyanobacteria) cell growth and an adaptation to increased light and potential oxidative stress (Rai, 2002; Nahar et al., 2015). Besides, these fatty acids enrichment could also help maintain osmotic pressure within cells and resist environmental stress. In autumn, the microbial community exhibited enrichment in pathways associated with transcription factors, amino acid metabolism, protein export and flagellar assembly, reflecting microbial adaptation to environmental changes and an accelerated rate of organic matter decomposition. Furthermore, despite the diversity and abundance of bacterioplankton communities in spring being lower than in autumn (Figure 3), microbial metabolic activity in spring was obviously higher than in autumn (Supplementary Figure 5).

## 4 Discussion

### 4.1 Analysis of nutrient sources in estuarine coastal areas

Important nutrient sources in coastal regions include not only seasonal external inputs but also the metabolic release from marine organisms (Baetge et al., 2021; Stephens et al., 2023; Bittner et al., 2024). In this study, nitrate, nitrite, and ammonium in seawater were significantly and negatively correlated with salinity (*P* < 0.05), highlighting that these nitrogen salts mainly originate from riverine inputs (Zhou et al., 2021). In summer and autumn, the concentrations of nitrate, nitrite, and ammonium were higher than that in spring due to increased surface runoff and river input. While salinity was lower in summer and autumn than that in spring due to increased freshwater input. Considering that phytoplankton are organic phosphorus sources, the negative correlation between chlorophyll *a* and phosphate (PO_4_^3–^) may indicate that inorganic phosphorus is actively absorbed and transformed into organic phosphorus where phytoplankton are abundant. Thus, this negative correlation might reflect the efficient biological utilization of phosphorus rather than a simple phosphorus deficiency. The negative correlation between chlorophyll *a* and TDP in Dongshan Beach suggests that bioavailable phosphorus may not fully correspond to TDP in the water. Phytoplankton growth might be limited by other factors like light, temperature and other nutrients, or phosphorus might be present in forms not directly usable by phytoplankton. Other studies in Qinhuangdao coastal waters found a significant positive correlation (*P* < 0.01) between total bacterioplankton abundance and TP, reinforcing the viability of TP as a key indicator for monitoring nutrient cycling influenced by human activity (He et al., 2017). Overall, the positive correlation between chlorophyll *a* and PON and POP supports the hypothesis that phytoplankton are significant sources of organic nitrogen and phosphorus in Western Beach. The negative correlation between phosphate and TDP might not directly indicate phosphorus deficiency but rather reflects the efficiency of phytoplankton absorption and utilization of inorganic phosphorus. These correlations emphasize the central role of primary producers in the carbon, nitrogen, and phosphorus cycles, and their response to changing environmental conditions. These results suggest that urban sewage discharge is an important nutrient input pathway in both beaches. Furthermore, in a recreational beach community, the installation of Best Management Practices (BMPs) systems can reduce TN loads by 86.9%, significantly decreasing nitrogen loading in estuarine waters (Grogan and Mallin, 2021). This previous study also demonstrates that rainfall-induced surface runoff is a significant source of nitrogen, while the impact on TP before and after the installation of the system was not significant (*P* < 0.05) (Grogan and Mallin, 2021). Therefore, to reduce phytoplankton bloom outbreaks in the study area, environmental agencies should enhance existing sewage treatment facilities, particularly by adding phosphorus removal steps.

### 4.2 Adaptive changes of bacterioplankton communities and their key environmental influencing factors

Seasonal change is a key factor driving the structural and functional succession of bacterioplankton communities. In spring, with rising temperatures and nutrient inputs from rainfall, certain microbial communities like *Persicirhabdus* and *Ulvibacter* from the phylum Bacteroidetes, as well as bacteria groups from Verrucomicrobiota, play essential roles in processing accumulated organic matter from winter, promoting the decomposition of high-molecular organic compounds (Sidhu et al., 2023). The diversity of these microbes and the presence of Patescibacteria demonstrate their adaptability to relatively low nutrient environments (Tian et al., 2020; Fujii et al., 2022). In summer, high temperatures and increased rainfall promote cyanobacterial blooms and the proliferation of specific microbes such as Planctomycetota, both playing crucial roles in decomposing algal polysaccharides produced by phytoplankton blooms and in nitrogen cycling (Sidhu et al., 2023). Additionally, predatory Bdellovibrionota and parasitic Chlamydiae also influence the microbial community structure (Li et al., 2021). In autumn, the decline in temperature and changes in nutrients directly impact microbial growth. Some members of the phylum Pseudomonadota, like HIMB11 and the OM60 (NOR5) clade, are particularly active in nutrient cycling to adapt to environmental changes. Moreover, the SAR324 clade and Marinimicrobia are significantly enriched, actively participating in carbon, nitrogen and sulfur cycling in shallow waters (Hawley et al., 2017; Boeuf et al., 2021). In autumn, abundant humic substances provide ample organic matter for microbes such as Myxococcota and Gemmatimonadota, enhancing their role in organic matter decomposition (Li et al., 2023; Mujakić et al., 2023). These seasonal changes and microbial activity adjustments reveal the functions of microbial communities in responding to environmental changes, maintaining ecological balance and supporting biogeochemical cycles. This is crucial to understanding how microbes adapt to environmental changes and their roles in the marine ecosystem.

Estuarine microbial communities play a critical role in global biogeochemical cycles. Planctomycetes are involved in the global nitrogen cycle, converting ammonia to nitrogen gas through anaerobic ammonia oxidation in nitrogen-limited and low-nutrient environments. This allows them to thrive in oligotrophic waters (Fuerst and Sagulenko, 2011). Groups of the phylum Chloroflexi play a key role in decomposing recalcitrant organic compounds like cellulose, and their enrichment at offshore stations indicates the accumulation and breakdown of complex organic matter (Speirs et al., 2019). Estuaries bring in large amounts of terrestrial organic matter and fluctuations in salinity and temperature, creating low-oxygen environments. Microbes such as Acidobacteriota, NB1-j, and SAR324 adapt to these conditions and participate in the carbon, sulfur, and nitrogen cycles (Boeuf et al., 2021; Flieder et al., 2021; Pushpakumara et al., 2023). Margulisbacteria groups are enriched at certain nearshore stations in both beaches in this study, although their ecological function in estuaries is not well understood. However, they are known to form symbiotic relationships with other microorganisms (Gruber-Vodicka et al., 2019). The diversity and succession characteristics of these microbial communities above highlight their importance in organic matter decomposition and element cycling in estuarine environments.

The bacterioplankton community is influenced by seasonal environmental changes, such as temperature, sunlight, rainfall, and salinity, as well as anthropogenic impacts, including coastal engineering, tourism activities, and port shipping (Kellogg et al., 2019; Wang C. et al., 2021; Wang H. et al., 2021; Doane et al., 2023). In the present study, the abundance and diversity of bacterioplankton communities in autumn are significantly higher than in spring and summer, likely related to environmental changes caused by plant and algal decay in late summer and early autumn (Li et al., 2020). These processes release substantial nutrients like carbon, nitrogen, and phosphorus, promoting microbial proliferation, abundant diversity, and nutrient cycling in estuaries (Crump and Bowen, 2024). For instance, the decomposition of marine plants provides abundant nutrients to support the proliferation of diverse microorganisms. Additionally, the mild temperatures of autumn might better support the coexistence of diverse microbial populations, enhancing community diversity, and evenness. In contrast, extreme summer temperatures may only allow the survival of a few highly adaptable species, thereby reducing microbial diversity in summer (Zilius et al., 2020).

Based on the box plot for alpha diversity analysis of bacterioplankton communities (Figure 3), the distribution of abundance, diversity, and evenness indices of the summer communities in the Western Beach is more dispersed, compared with that in Dongshan Beach. This suggests notable structural differences of bacterioplankton communities among stations, possibly due to geographic factors and more complex environmental influences. This area contains the estuaries of two urban rivers, the Datanghe and Xiaotanghe rivers, as well as a man-made island nearshore stations. Urban sewage carries large amounts of nutrients such as nitrogen and phosphorus into the study area. Due to poor hydrodynamic conditions, the rivers carry substantial pollutants with weak exchange capacity with offshore waters. Additionally, the high number of summer tourists in the Western Beach increases urea discharge, further elevating nitrogen concentration. These factors result in more complex environmental conditions in the nearshore waters of Western Beach during the summer. Similar phenomena have been observed in the Chesapeake Bay with high human activity. Riverine inputs resulting in eutrophication and increased organic pollutants have promoted the growth of heterotrophic Proteobacteria (such as Deltaproteobacteria and Gammaproteobacteria) and Bacteroidetes (Flavobacteria) (Wang H. et al., 2021).

In this study, season was the key factor dominating the spatiotemporal distribution of bacterioplankton communities. While investigations ranging from the Zhoushan Sea area near the Yangtze River estuary to the continental shelf edge revealed that bacterioplankton distribution is influenced by the combined effects of temperature and offshore distance (Wang et al., 2023). The substantial environmental changes between spring and summer lead to pronounced differences in their composition. In this study, the significant effect of seawater salinity on the spring bacterioplankton community was similar to findings from the Pearl River Estuary in autumn (Mai et al., 2020). Spring is generally cooler, with gradually increasing sunlight and rainfall and rising water temperatures, accelerating microbial growth and metabolic activity. In summer, warm temperatures, intense sunlight, increased rainfall, surface runoff, tourism, and shipping significantly raise coastal pollution from estuarine inputs, greatly stimulating bacterioplankton proliferation and metabolic activity. In autumn, despite lower water temperatures and reduced sunlight, the decline in pollutants from riverine input and tourism coincides with abundant organic matter accumulated during summer, as well as nutrient releases from the decomposition of dead plankton. These conditions promote prolific bacterioplankton growth. This phenomenon is also observed at the late stage of algal blooms, where the γ-Proteobacteria SAR86 clade and OM43 groups become enriched (Sidhu et al., 2023). These factors contribute to the frequent algae blooms in the study area during summer and autumn, especially due to the substantial increase in nitrogen levels in summer. Additionally, environmental drivers of bacterioplankton vary among different marine regions. For instance, even in regions with similar climatic and geographical conditions, such as a semi-closed bay on the southern coast of South Korea, Verrucomicrobiota only showed a significant positive correlation (*P* < 0.05) with phytoplankton abundance, while its correlation with salinity, temperature, DOC and Chl *a* was not significant (*P* > 0.05) (Kim et al., 2016). While, in the present study, Verrucomicrobiota exhibited a significant positive correlation with salinity in the western of Bohai Bay (*P* < 0.05). These results suggest that bacterioplankton communities in estuaries may also be influenced by other factors, such as heavy metals, microplastics, antibiotics, and other pollutants input through human activities (Yang et al., 2015; Xu et al., 2020; Yi et al., 2021; Zheng et al., 2021). Besides, future research should devote more effort to revealing the combined effects of multiple factors.

### 4.3 Metabolic changes of bacterioplankton communities

The metabolic characteristics of bacterioplankton communities respond to seasonal environmental changes, influencing nutrient cycling, and energy metabolism in the marine ecosystem. The metabolic features of the spring bacterioplankton community are closely related to the increased photosynthesis activity, which directly responds to the increased sunlight and temperature in spring, playing a crucial role in enhancing ocean primary productivity. The summer metabolic activities reflect the microbial adaptation to the energy- and nutrient-rich environment, possibly due to increased tourism and nutrient input during the summer. In autumn, changes in microbial metabolic activities reveal how these communities accelerate organic matter decomposition to adapt to lower temperatures and changes in nutrient sources, thus coping with the environmental stress brought about by temperature drops. These changes demonstrate the adaptive strategies of microbial communities in nutrient cycling, energy metabolism, managing environmental stress, as well as maintaining cellular structure and function. And these closely linked to seasonal environmental changes and the resultant shifts in ecosystem functions. Understanding these metabolic pathway changes helps us comprehend how microbial communities respond to seasonal environmental fluctuations and their impact on the overall health and stability of the ecosystem.

In the present study, we observed significant enrichment of lysine and other essential amino acid synthesis pathways as well as glutathione metabolism in summer samples, which may be related to the significant increase in cyanobacteria. The enhanced synthesis of these amino acids is closely related to the defense mechanisms of cells under light exposure and oxidative stress (Rai, 2002; Nahar et al., 2015). Although the specific roles of these mechanisms in cyanobacteria are not yet fully understood, it is reasonable to speculate that the enrichment of these pathways may contribute to the defense responses of cyanobacteria in environments with high light intensity and potentially increased oxidative stress during summer. Notably, although proline is usually more closely associated with stress responses (Verbruggen and Hermans, 2008; Dar et al., 2016; Ingrisano et al., 2023), no significant changes in its biosynthesis pathways were observed in this study. This may indicate the existence of unique mechanisms in cyanobacteria that differ from the known stress response pathways in plants. Additionally, the increased synthesis of these amino acids may help bacterioplankton maintain intracellular osmotic pressure, resist environmental stress, and maintain cell functionality and structure. Given the current scarcity of reference on the roles of lysine and other amino acids in cyanobacteria, future research should focus on the expression patterns and physiological functions of these amino acid pathways under various environmental stresses. Particularly, studies should explore their specific roles in the photosynthetic activity and oxidative defense of cyanobacteria.

On the whole, this study investigated the spatiotemporal variations of bacterioplankton communities and the environmental driving factors of coastal waters of two tourist beaches in Qinhuangdao. However, due to the absence of winter samples in the survey voyages, only the bacterial communities and environmental characteristics of spring, summer, and autumn were analyzed. In other studies of the coastal waters of Qinhuangdao, TN, temperature, Chl *a*, and DO were the most crucial factors in differentiating the samples from warm and cold seasons (He et al., 2017). The abundance of α-Proteobacteria, Firmicutes, and Actinobacteria in winter (December) was significantly lower than in summer and spring (He et al., 2017). Additionally, DO and salinity were key environmental factors influencing the bacterioplankton abundance. The study area is located in a semi-enclosed bay, and the bacterioplankton communities show significant differences compared to those in more open Chinese waters. In the eastern East China Sea, the bacterioplankton community of surface seawater in summer is predominantly composed of Firmicutes (85.9%), Actinobacteria (8.1%), and Proteobacteria (5.4%) (Wang et al., 2023). Considering that all sampling sites in this study are close to the coastline, it is challenging to compare the bacterioplankton communities with those in offshore areas. Therefore, further expanding the sampling range and duration will provide a deeper understanding of the temporal and spatial variations of bacterioplankton communities as well as their environmental driving factors in the offshore waters of Qinhuangdao.

## 5 Conclusion

This study systematically analyzed the bacterioplankton communities in the coastal waters of Western Beach and Dongshan Beach in Qinhuangdao, revealing structural and functional changes in these communities under seasonal variations and human activities. In spring, with rising temperatures and increased precipitation, specific microorganisms like Bacteroidetes and Verrucomicrobiota excelled in organic matter decomposition, while enhanced photosynthesis boosted primary productivity. In summer, high temperatures, river inputs and increased tourism activity intensified nutrient inputs, promoting the growth of photosynthetic microorganisms such as Cyanobacteria. And these environmental changes are closely linked to phytoplankton blooms in this study area. In autumn, the microbial communities adapted to the temperature decline, accelerating organic matter decomposition and displaying strong environmental adaptability and functional diversity. Additionally, spatial distribution analysis showed that microbial community structure is significantly influenced by local environmental conditions, with urban sewage discharge and coastal engineering significantly affecting nutrient dynamics. These findings emphasize the importance of enhancing wastewater treatment and maintaining favorable coastal hydrodynamic conditions to prevent outbreaks of the phytoplankton bloom. However, the sample collection was limited to the year 2021, which somewhat restricts our ability to comprehensively assess long-term trends in seasonal variation. Future studies will need to involve data collection over several years to further validate our findings. We believe this will provide deeper insights into the seasonal variation of bacterioplankton communities in this region. This study not only deepens our understanding of marine microbial community dynamics but also underscores the core role of bacterioplankton in global biogeochemical cycles, providing a scientific basis for future management and conservation of marine ecosystem.

## Data availability statement

The datasets presented in this study can be found in online repositories. The names of the repository/repositories and accession number(s) can be found at: https://www.ncbi.nlm.nih.gov/, PRJNA1111961.

## Author contributions

QW: Conceptualization, Formal analysis, Investigation, Methodology, Project administration, Visualization, Writing – original draft, Writing – review & editing. JY: Conceptualization, Funding acquisition, Methodology, Writing – review & editing. XL: Investigation, Visualization, Writing – review & editing. YZ: Conceptualization, Investigation, Methodology, Project administration, Visualization, Writing – original draft. JZ: Methodology, Project administration, Writing – review & editing. JW: Formal analysis, Funding acquisition, Writing – review & editing. JM: Visualization, Writing – original draft. XY: Formal analysis, Writing – original draft. RH: Formal analysis, Writing – original draft.

## Footnotes

1
https://qiime2.org/

2
https://www.omicstudio.cn
