# Supplementary material for: Seasonal and anthropogenic influences on bacterioplankton communities: ecological impacts in the coastal waters of Qinhuangdao, Northern China
Source: Front Microbiol. 2024 Jun 19;15:1431548. doi: 10.3389/fmicb.2024.1431548 (PMC11220261; doi:10.3389/fmicb.2024.1431548)
Supplement: Supplementary file 1 [file Data_Sheet_1.docx]

Supplementary Material

**A**


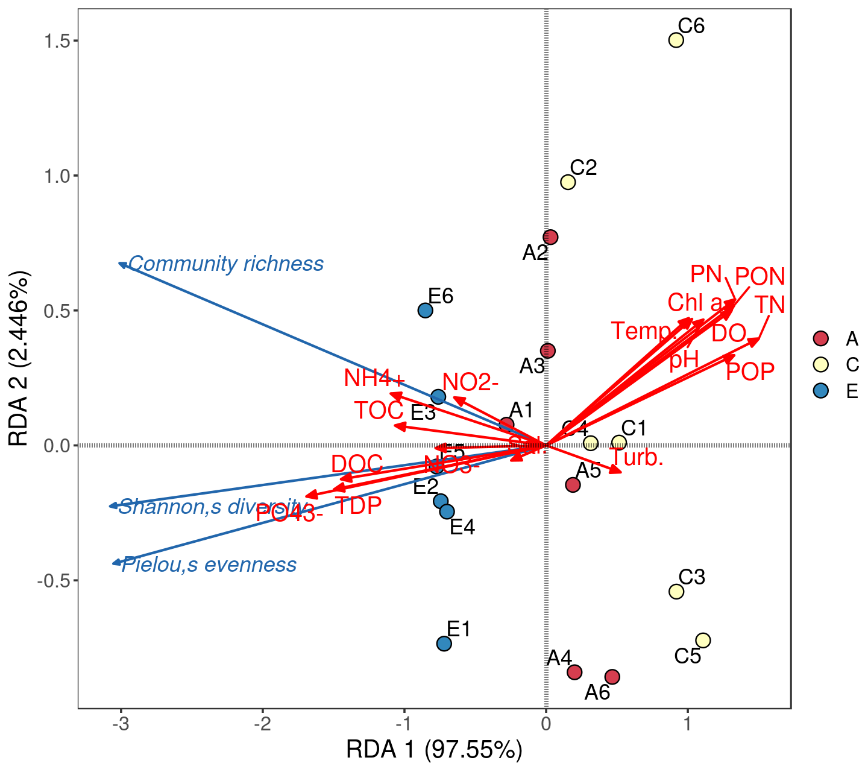


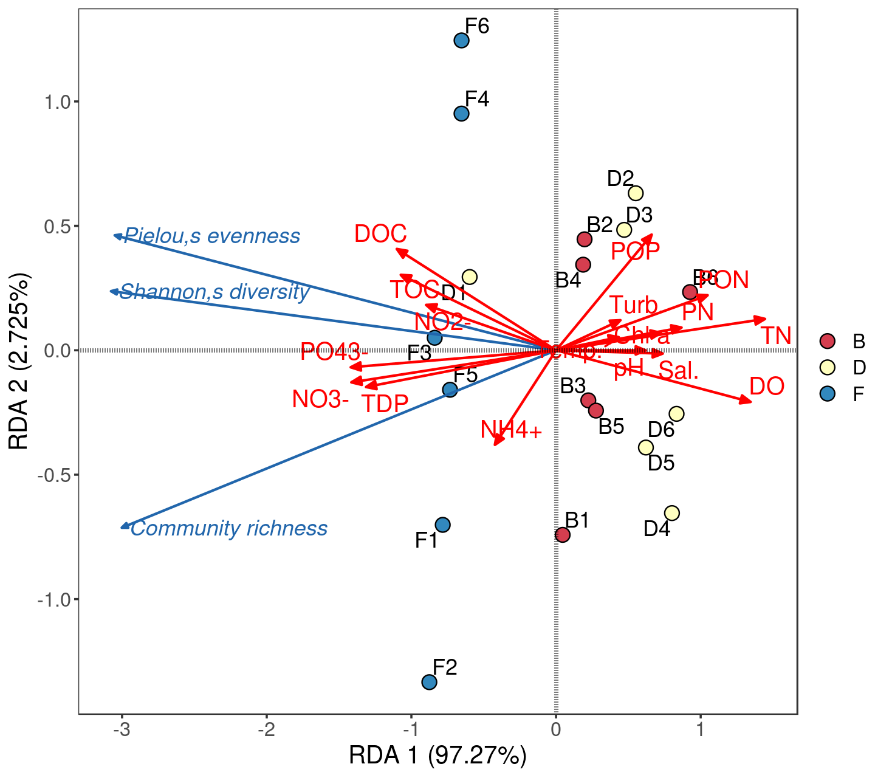


**B**

**Supplementary** **Figure 1.** Redundancy analysis (RDA) between diversity indicators and environment parameters in the seawater of Western Beach (A) and Dongshan Beach (B). Capital letters with different colored circles represent sampling information (A: Western Beach in spring; B: Dongshan Beach in spring; C: Western Beach in summer; D: Dongshan Beach in summer; E: Western Beach in autumn; F: Dongshan Beach in autumn).


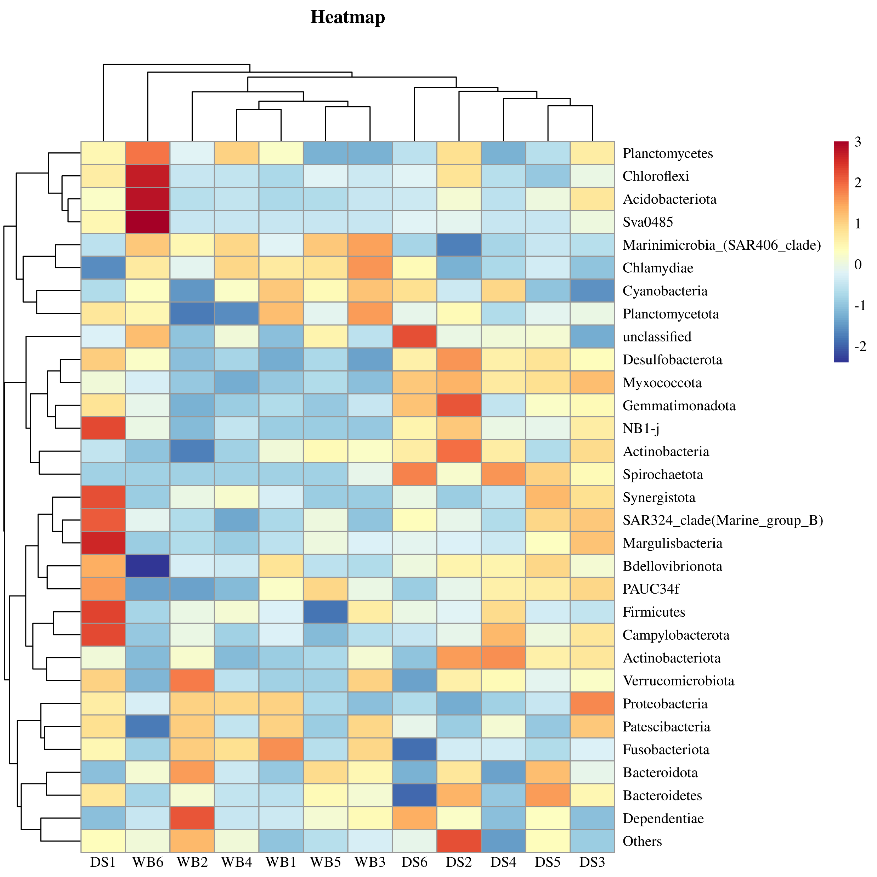


**A**

**B**


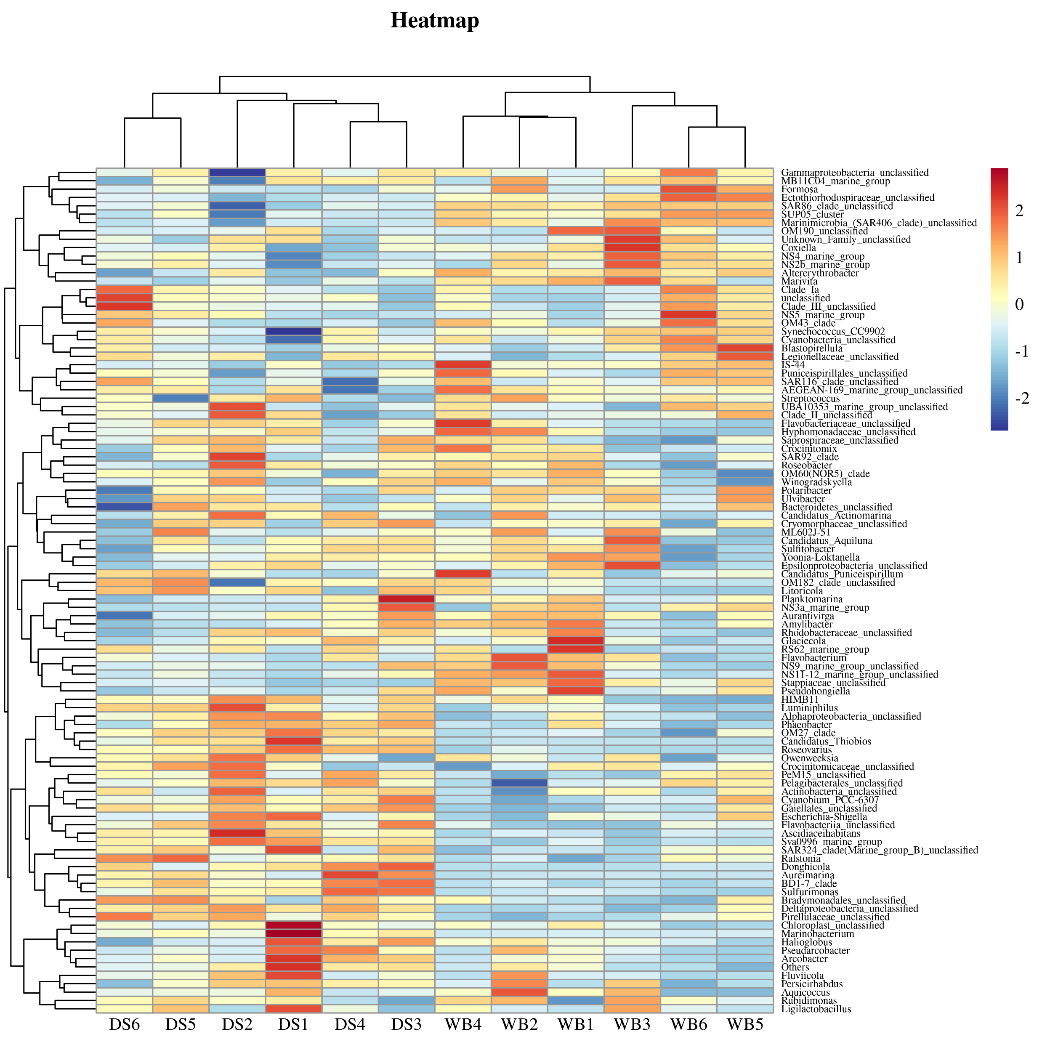


**Supplementary Figure 2.** Correlation heatmap of bacteriophankton communities in different stations of Western Beach (WB) and Dongshan Beach (DB) in the present study with top 30 phylum (A) and top 100 genera (B).


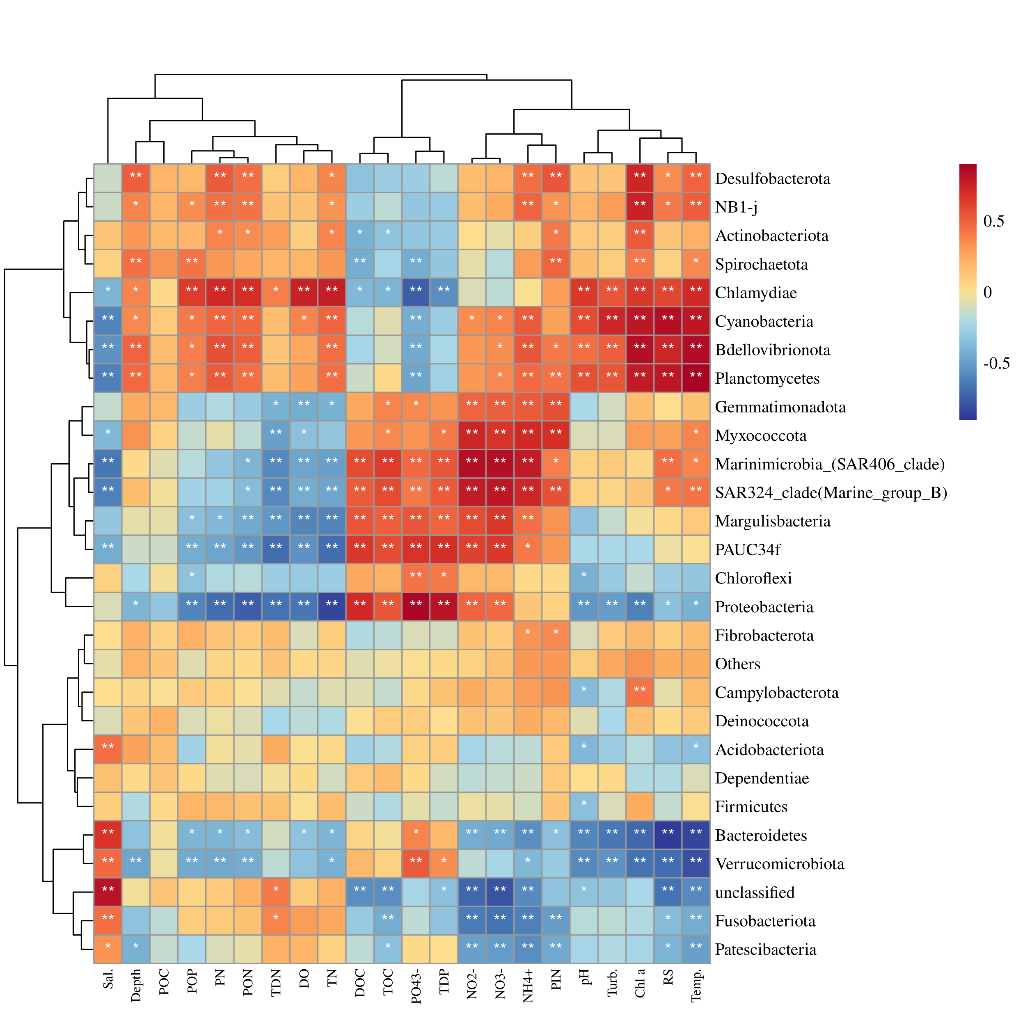


**A**


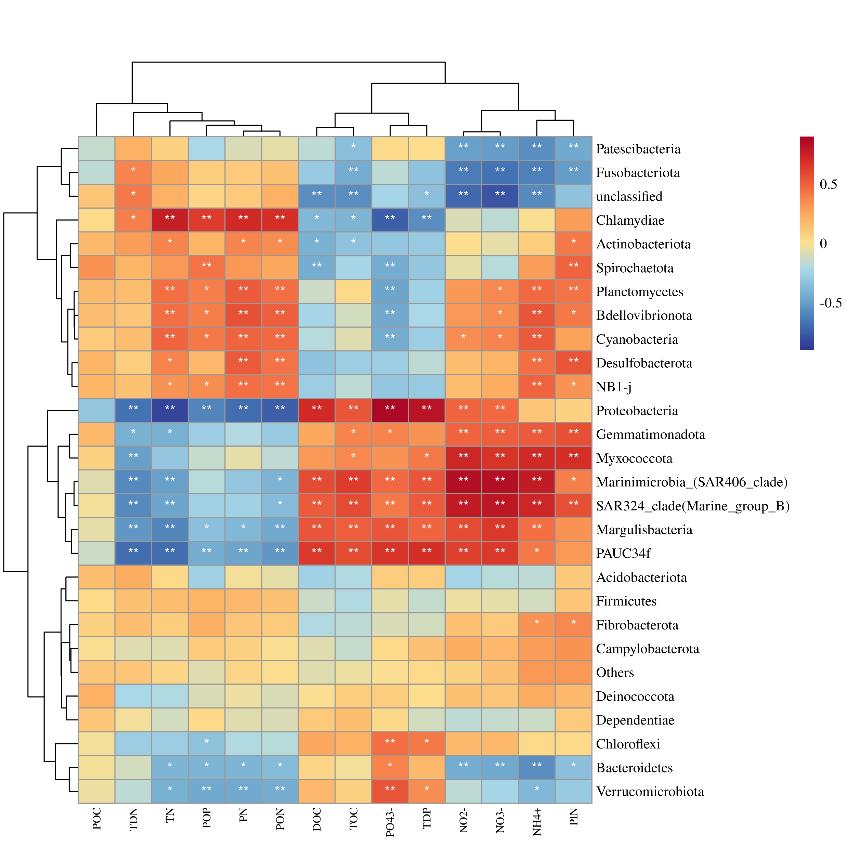


**B**

**Supplementary Figure 3.** Spearman correlation heatmap of the environment parameters and bacteriophankton communities at the phylum (A) and genus (B) levels with top 28 in two beaches of the present study.


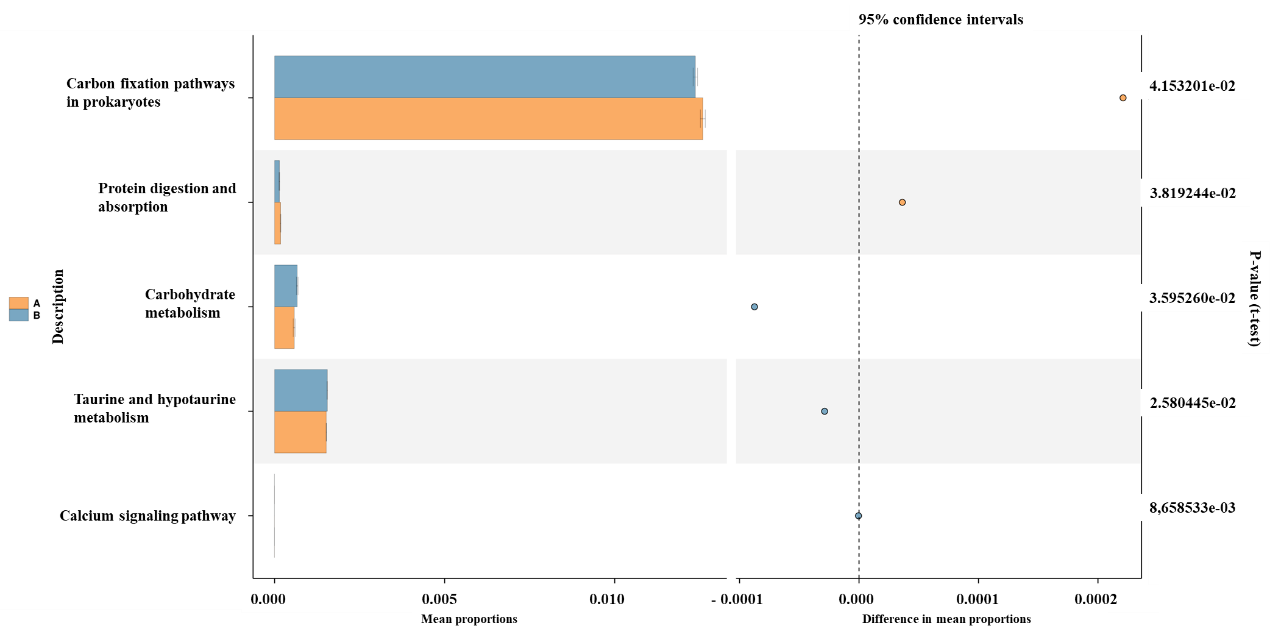


**A**


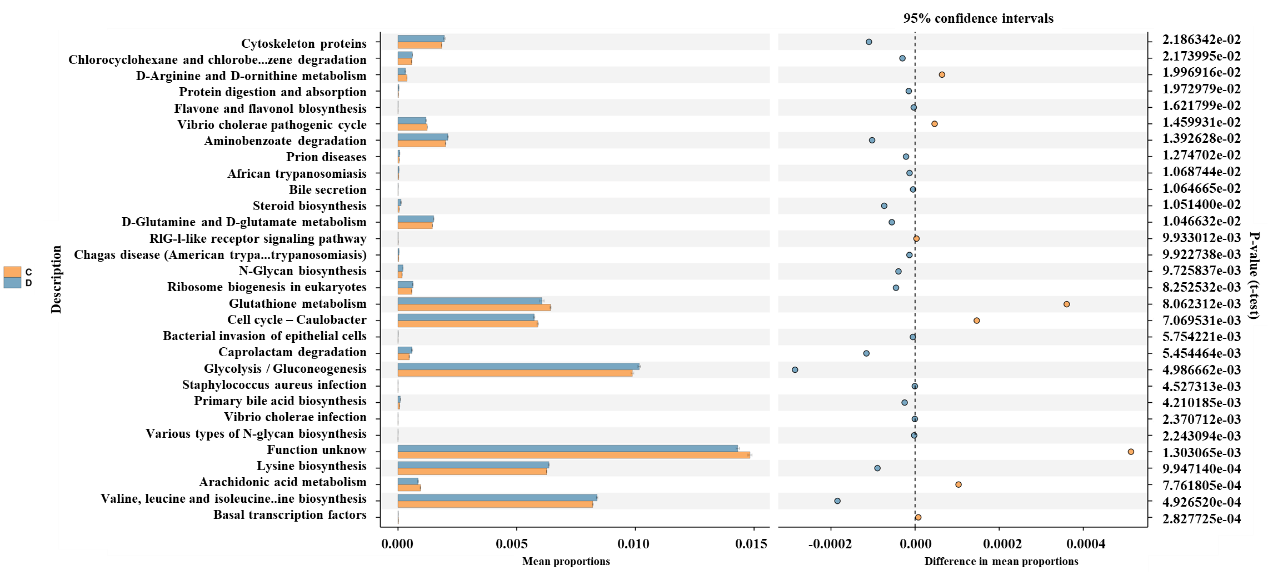


**B**


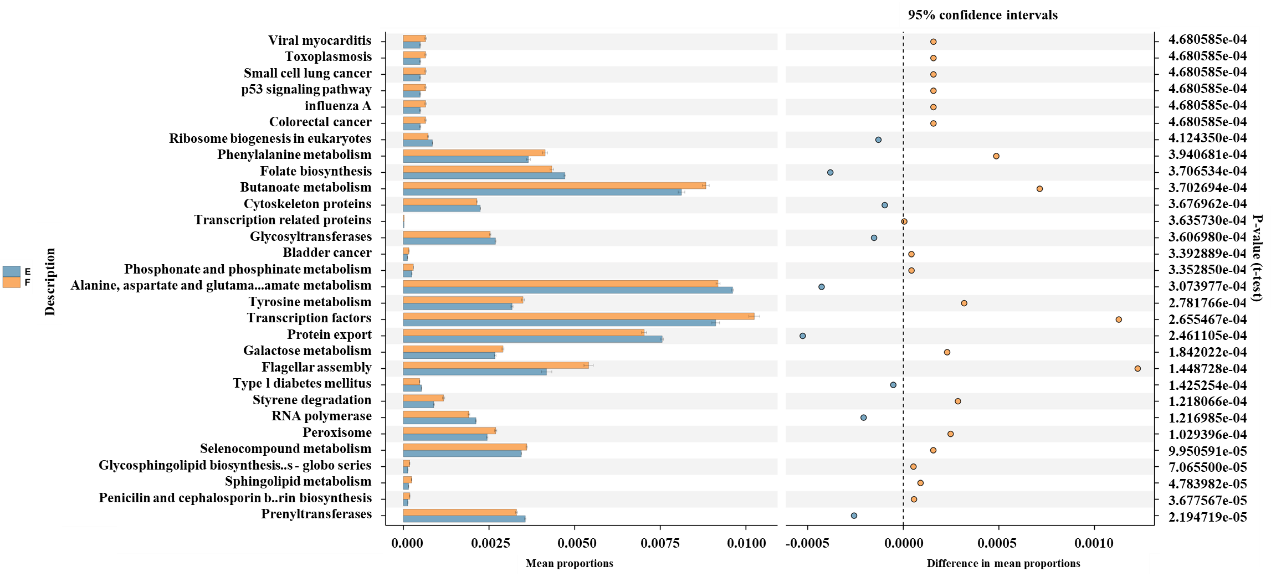


**C**

**Supplementary Figure 4.** The top 30 functional predictions of bacteriophankton communities with significant differences (*P* < 0.05) at KEGG level 3 in April (A), August (B) and October (C).


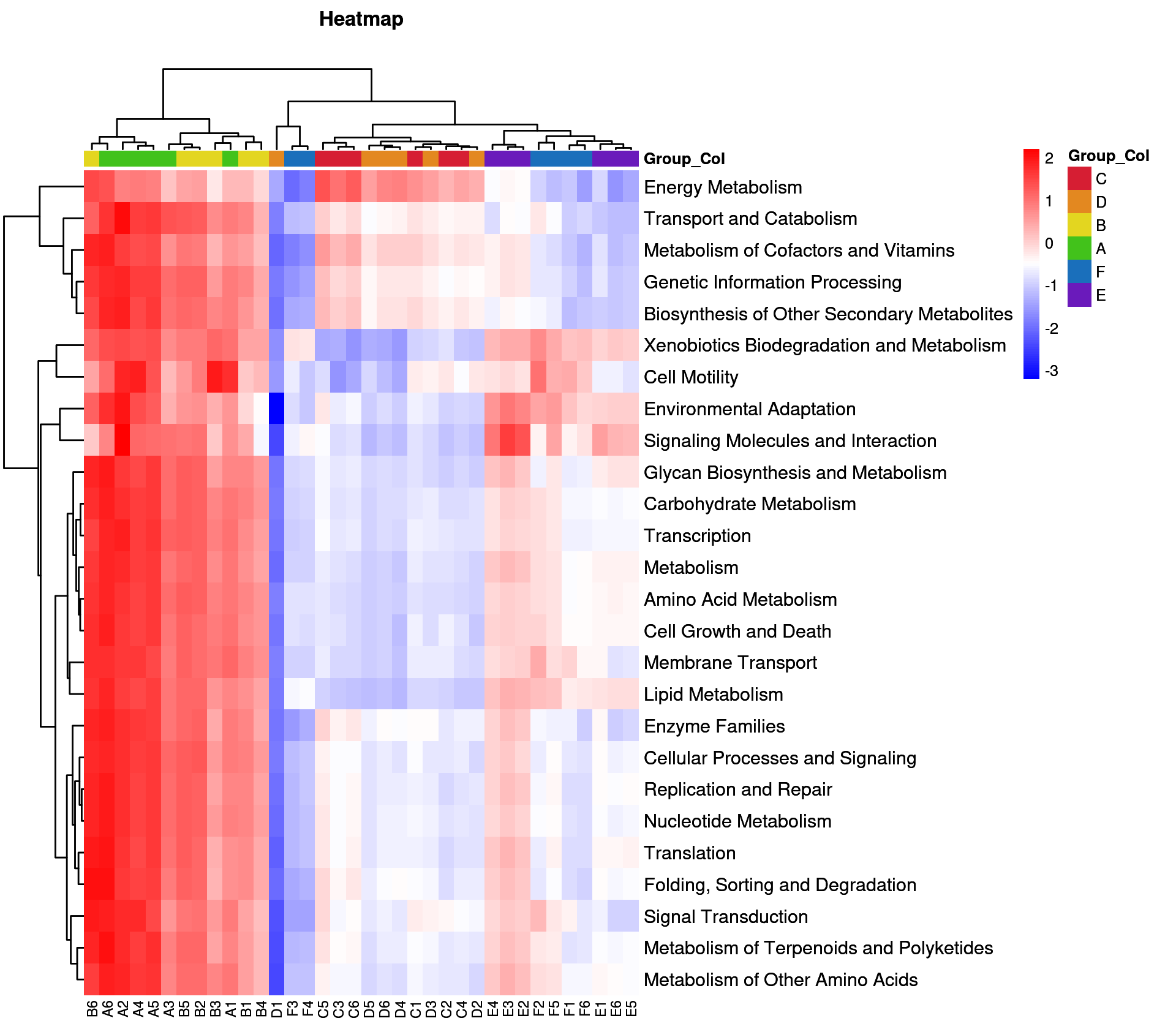


**Supplementary Figure 5.** Cluster heatmap of predicted functions at KEGG level 2 of the bacteriophankton communities across different stations. Capital letters in the sample name mean sampling information (A: Western Beach in spring; B: Dongshan Beach in spring; C: Western Beach in summer; D: Dongshan Beach in summer; E: Western Beach in autumn; F: Dongshan Beach in autumn).

**TABLE S1 Sapling information and station labels in different seasons.**

| **Sampling area** | **Station** | **Longitude (ºE)** | **Latitude (ºN)** | **Station label** | | |
| --- | --- | --- | --- | --- | --- | --- |
|  |  |  |  | **April** | **August** | **October** |
| Western Beach | 1 | 119.558830 | 39.893419 | A1 | C1 | E1 |
|  | 2 | 119.570005 | 39.878961 | A2 | C2 | E2 |
|  | 3 | 119.583767 | 39.879713 | A3 | C3 | E3 |
|  | 4 | 119.542289 | 39.868657 | A4 | C4 | E4 |
|  | 5 | 119.562390 | 39.867961 | A5 | C5 | E5 |
|  | 6 | 119.569871 | 39.856680 | A6 | C6 | E6 |
| Dongshan Beach | 1 | 119.637828 | 39.909599 | B1 | D1 | F1 |
|  | 2 | 119.636568 | 39.896166 | B2 | D2 | F2 |
|  | 3 | 119.635021 | 39.880355 | B3 | D3 | F3 |
|  | 4 | 119.621636 | 39.897011 | B4 | D4 | F4 |
|  | 5 | 119.616085 | 39.884406 | B5 | D5 | F5 |
|  | 6 | 119.604980 | 39.888089 | B6 | D6 | F6 |

**TABLE S2 Environmental parameters of seawater.**

| **Parameter** | **A1** | **A2** | **A3** | **A4** | **A5** | **A6** | **B1** | **B2** | **B3** | **B4** | **B5** | **B6** |
| --- | --- | --- | --- | --- | --- | --- | --- | --- | --- | --- | --- | --- |
| Depth (m) | 5.6 | 7.7 | 8.0 | 6.4 | 8.6 | 8.1 | 9.0 | 6.9 | 8.0 | 7.0 | 9.0 | 9.8 |
| pH | 8.06 | 8.13 | 8.00 | 8.02 | 8.06 | 8.07 | 7.82 | 7.98 | 7.87 | 8.01 | 8.17 | 8.02 |
| Temperature (℃) | 13.2 | 13.0 | 12.6 | 12.2 | 12.0 | 11.8 | 11.4 | 11.2 | 11.7 | 11.4 | 11.2 | 11.6 |
| Salinity | 32.169 | 32.374 | 32.406 | 32.438 | 32.406 | 32.868 | 32.485 | 32.597 | 32.620 | 32.404 | 32.834 | 32.740 |
| DO (mg/L) | 10.30 | 9.24 | 9.52 | 9.58 | 9.70 | 9.54 | 10.70 | 9.28 | 10.20 | 10.00 | 8.74 | 10.80 |
| NO_2_^-^（mg/L） | 0.0034 | 0.0018 | 0.0015 | 0.0015 | 0.0012 | 0.0012 | 0.0021 | 0.0016 | 0.0042 | 0.0525 | 0.0020 | 0.0013 |
| NO_3_^-^（mg/L） | 0.0643 | 0.0338 | 0.0139 | 0.0323 | 0.0160 | 0.0363 | 0.0829 | 0.0356 | 0.0597 | 0.0835 | 0.0449 | 0.0430 |
| NH_4_^+^（mg/L） | 0.0328 | 0.0162 | 0.0183 | 0.0140 | 0.0164 | 0.0176 | 0.0569 | 0.0266 | 0.0180 | 0.0218 | 0.0185 | 0.0134 |
| PO_4_^3-^（mg/L） | 0.0072 | 0.0050 | 0.0047 | 0.0033 | 0.0044 | 0.0047 | 0.0126 | 0.0053 | 0.0047 | 0.0067 | 0.0053 | 0.0050 |
| Reactive Silicate（mg/L） | 0.1490 | 0.1140 | 0.0964 | 0.0964 | 0.1140 | 0.0906 | 0.1080 | 0.0906 | 0.2500 | 0.3690 | 0.1310 | 0.2060 |
| Turbidity（NTU） | 4.94 | 4.12 | 3.94 | 4.78 | 5.23 | 3.14 | 4.51 | 3.15 | 2.14 | 4.67 | 3.74 | 4.85 |
| Chl a (µg/L) | 3.17 | 2.70 | 3.12 | 2.10 | 1.44 | 0.92 | 5.03 | 2.52 | 5.69 | 4.61 | 1.96 | 3.08 |
| TOC (mg/L) | 4.6450 | 6.1145 | 4.5575 | 5.6443 | 6.0560 | 4.8835 | 4.2805 | 6.2153 | 4.2715 | 4.3880 | 5.1885 | 4.8690 |
| DOC (mg/L) | 4.3655 | 4.1388 | 4.2735 | 4.0018 | 4.2653 | 4.2620 | 2.1435 | 4.7573 | 4.0765 | 3.7583 | 3.3575 | 3.8140 |
| POC (mg/L) | 0.2795 | 1.9758 | 0.2840 | 1.6425 | 1.7908 | 0.6215 | 2.1370 | 1.4580 | 0.1950 | 0.6298 | 1.8310 | 1.0550 |
| TN (μg/L) | 145.7778 | 174.6667 | 149.1111 | 171.3333 | 148.0000 | 115.7778 | 219.1111 | 150.2222 | 173.5556 | 245.7778 | 149.1111 | 181.3333 |
| TDN (μg/L) | 121.3333 | 105.7778 | 96.8889 | 104.6667 | 98.0000 | 89.1111 | 90.2222 | 116.8889 | 143.5556 | 140.2222 | 126.8889 | 144.6667 |
| PN (μg/L) | 28.3333 | 75.0000 | 41.6667 | 66.6667 | 50.0000 | 26.6667 | 128.8889 | 33.3333 | 30.0000 | 105.5556 | 26.6667 | 37.7778 |
| PIN (μg/L) | 8.7156 | 11.6600 | 17.3526 | 7.4723 | 1.3217 | 3.8081 | 4.7242 | 1.5835 | 5.9020 | 7.7341 | 3.8081 | 8.1267 |
| PON (μg/L) | 19.6178 | 63.3400 | 24.3141 | 59.1943 | 48.6783 | 22.8585 | 124.1647 | 31.7499 | 24.0980 | 97.8215 | 22.8585 | 29.6511 |
| TP (μg/L) | / | / | / | / | / | / | / | / | / | / | / | / |
| TDP (μg/L) | 6.9677 | 7.9355 | 6.4839 | 6.4839 | 6.7527 | 6.4301 | 15.7849 | 7.2903 | 13.3118 | 10.6237 | 7.6129 | 12.7742 |
| PP (μg/L) | / | / | / | / | / | / | / | / | / | / | / | / |
| POP (μg/L) | 2.8068 | 4.6607 | 2.2088 | 3.9431 | 2.4480 | 3.6042 | 0.9728 | 4.6208 | 1.7901 | 6.3153 | 0.5741 | 0.9329 |
| PIP (μg/L) | / | / | / | / | / | / | / | / | / | / | / | / |
| **Parameter** | **C1** | **C2** | **C3** | **C4** | **C5** | **C6** | **D1** | **D2** | **D3** | **D4** | **D5** | **D6** |
| Depth (m) | 6.5 | 9.1 | 9.3 | 8.4 | 9.6 | 9.8 | 8.2 | 9.9 | 9.9 | 9.0 | 15.8 | 9.0 |
| pH | 8.46 | 8.44 | 8.27 | 8.48 | 8.38 | 8.32 | 7.92 | 8.10 | 8.14 | 8.16 | 8.15 | 8.16 |
| Temperature (℃) | 27.4 | 27.2 | 26.8 | 27.0 | 26.8 | 26.8 | 26.3 | 26.2 | 26.0 | 26.8 | 26.8 | 27.0 |
| Salinity | 28.027 | 28.013 | 25.774 | 28.522 | 28.892 | 29.675 | 30.104 | 30.041 | 29.953 | 29.795 | 29.741 | 29.518 |
| DO (mg/L) | 12.10 | 12.10 | 11.30 | 12.30 | 11.10 | 11.50 | 8.22 | 9.94 | 9.83 | 10.40 | 10.10 | 10.70 |
| NO_2_^-^（mg/L） | 0.0194 | 0.0194 | 0.0248 | 0.0120 | 0.0210 | 0.0234 | 0.0385 | 0.0228 | 0.0275 | 0.0222 | 0.0170 | 0.0308 |
| NO_3_^-^（mg/L） | 0.0641 | 0.0673 | 0.0963 | 0.0727 | 0.0871 | 0.0907 | 0.1560 | 0.0540 | 0.0845 | 0.0838 | 0.0721 | 0.0772 |
| NH_4_^+^（mg/L） | 0.0256 | 0.0446 | 0.0375 | 0.0334 | 0.0322 | 0.0385 | 0.0888 | 0.0781 | 0.0771 | 0.0592 | 0.0880 | 0.0654 |
| PO_4_^3-^（mg/L） | 0.0022 | 0.0025 | 0.0022 | 0.0028 | 0.0022 | 0.0025 | 0.0039 | 0.0028 | 0.0034 | 0.0013 | 0.0022 | 0.0011 |
| Reactive Silicate（mg/L） | 1.6020 | 1.6020 | 2.2010 | 1.3180 | 1.6860 | 1.4790 | 0.7700 | 0.5380 | 0.4930 | 0.3770 | 0.5250 | 0.4610 |
| Turbidity（NTU） | 5.74 | 4.68 | 6.45 | 6.52 | 7.65 | 7.35 | 5.74 | 4.65 | 7.24 | 5.13 | 6.42 | 4.89 |
| Chl a (µg/L) | 14.80 | 11.60 | 9.29 | 16.60 | 9.93 | 10.30 | 45.00 | 32.00 | 32.70 | 39.00 | 29.40 | 28.20 |
| TOC (mg/L) | 5.1460 | 5.2450 | 5.2428 | 5.5585 | 5.0825 | 5.7728 | 5.0868 | 5.4578 | 5.3738 | 4.8730 | 4.8038 | 4.9705 |
| DOC (mg/L) | 4.5348 | 4.0810 | 4.0580 | 4.3628 | 4.2245 | 4.3355 | 4.0128 | 3.6408 | 3.3255 | 3.6668 | 3.4673 | 3.0535 |
| POC (mg/L) | 0.6113 | 1.1640 | 1.1848 | 1.1958 | 0.8580 | 1.4373 | 1.0740 | 1.8170 | 2.0483 | 1.2063 | 1.3365 | 1.9170 |
| TN (μg/L) | 255.7778 | 232.4444 | 271.3333 | 298.0000 | 266.8889 | 248.0000 | 200.2222 | 241.3333 | 242.4444 | 210.2222 | 253.5556 | 258.0000 |
| TDN (μg/L) | 106.2222 | 87.3333 | 205.1111 | 105.1111 | 112.8889 | 137.3333 | 245.6667 | 118.4444 | 140.6667 | 114.0000 | 148.4444 | 92.8889 |
| PN (μg/L) | 149.5556 | 145.1111 | 66.2222 | 192.8889 | 154.0000 | 110.6667 | 88.4444 | 122.8889 | 101.7778 | 96.2222 | 105.1111 | 165.1111 |
| PIN (μg/L) | 7.3415 | 7.0798 | 8.1267 | 9.8279 | 9.6970 | 14.2773 | 7.8649 | 10.4822 | 13.8847 | 11.3983 | 17.4180 | 17.0254 |
| PON (μg/L) | 142.2141 | 138.0314 | 58.0956 | 183.0610 | 144.3030 | 96.3894 | 80.5795 | 112.4067 | 87.8931 | 84.8240 | 87.6931 | 148.0857 |
| TP (μg/L) | 39.1778 | 31.6222 | 38.5111 | 36.6222 | 25.6222 | 31.1778 | 36.2889 | 32.9556 | 39.5111 | 39.8444 | 42.6222 | 35.4000 |
| TDP (μg/L) | 7.0667 | 9.1778 | 6.9556 | 6.8444 | 6.1778 | 5.6222 | 5.7333 | 5.6222 | 7.9556 | 7.0667 | 5.9556 | 6.0667 |
| PP (μg/L) | 32.1111 | 22.4444 | 31.5556 | 29.7778 | 19.4444 | 25.5556 | 30.5556 | 27.3333 | 31.5556 | 32.7778 | 36.6667 | 29.3333 |
| POP (μg/L) | 5.3584 | 4.1823 | 4.8003 | 4.4614 | 3.7039 | 4.3617 | 4.9597 | 4.6607 | 5.9764 | 5.7173 | 5.9565 | 5.3584 |
| PIP (μg/L) | 26.7527 | 18.2622 | 26.7553 | 25.3164 | 15.7406 | 21.1939 | 25.5958 | 22.6726 | 25.5792 | 27.0605 | 30.7102 | 23.9749 |
| **Parameter** | **E1** | **E2** | **E3** | **E4** | **E5** | **E6** | **F1** | **F2** | **F3** | **F4** | **F5** | **F6** |
| Depth (m) | 5.1 | 8.0 | 7.6 | 5.3 | 8.5 | 8.5 | 6.0 | 8.9 | 9.8 | 8.4 | 9.6 | 9.5 |
| pH | 8.06 | 8.08 | 8.10 | 8.07 | 8.06 | 8.05 | 8.05 | 8.05 | 8.02 | 8.02 | 8.03 | 8.05 |
| Temperature (℃) | 14.8 | 15.2 | 15.2 | 14.6 | 14.8 | 15.0 | 15.0 | 15.0 | 15.6 | 15.6 | 15.6 | 15.2 |
| Salinity | 29.352 | 29.608 | 29.229 | 29.089 | 29.386 | 29.573 | 29.393 | 29.627 | 29.458 | 29.419 | 29.497 | 29.585 |
| DO (mg/L) | 8.68 | 9.20 | 9.32 | 8.81 | 9.28 | 9.10 | 8.31 | 8.90 | 9.58 | 9.09 | 9.32 | 8.80 |
| NO_2_^-^（mg/L） | 0.0502 | 0.0482 | 0.0493 | 0.0527 | 0.0526 | 0.0515 | 0.0458 | 0.0456 | 0.0447 | 0.0471 | 0.0462 | 0.0462 |
| NO_3_^-^（mg/L） | 0.1460 | 0.1510 | 0.1600 | 0.1640 | 0.1610 | 0.1760 | 0.1700 | 0.1540 | 0.1130 | 0.1570 | 0.1410 | 0.1480 |
| NH_4_^+^（mg/L） | 0.0801 | 0.0540 | 0.0662 | 0.0646 | 0.1010 | 0.1000 | 0.0858 | 0.0672 | 0.0517 | 0.0582 | 0.0437 | 0.0318 |
| PO_4_^3-^（mg/L） | 0.0140 | 0.0115 | 0.0126 | 0.0143 | 0.0171 | 0.0138 | 0.0129 | 0.0132 | 0.0107 | 0.0083 | 0.0096 | 0.0098 |
| Reactive Silicate（mg/L） | 0.4750 | 0.4870 | 0.5240 | 0.4870 | 0.4870 | 0.5240 | 0.3580 | 0.3460 | 0.3060 | 0.3360 | 0.2900 | 0.3270 |
| Turbidity（NTU） | 7.23 | 6.14 | 5.71 | 4.89 | 4.51 | 3.74 | 4.51 | 4.23 | 3.74 | 4.67 | 3.25 | 4.17 |
| Chl a (µg/L) | 3.25 | 3.19 | 3.24 | 1.98 | 2.09 | 1.03 | 4.26 | 4.24 | 2.85 | 4.41 | 3.79 | 6.08 |
| TOC (mg/L) | 6.6773 | 6.3485 | 6.3970 | 6.3745 | 6.1575 | 6.2718 | 5.7580 | 6.1988 | 6.4585 | 5.4878 | 6.0948 | 6.0975 |
| DOC (mg/L) | 5.5958 | 4.9908 | 5.6945 | 5.5235 | 5.1443 | 5.3635 | 4.5713 | 5.2363 | 4.9370 | 4.5840 | 4.5860 | 5.1023 |
| POC (mg/L) | 1.0815 | 1.3578 | 0.7025 | 0.8510 | 1.0133 | 0.9083 | 1.1868 | 0.9625 | 1.5215 | 0.9038 | 1.5088 | 0.9953 |
| TN (μg/L) | 100.6511 | 110.2150 | 102.0218 | 103.0810 | 129.6542 | 113.7040 | 98.3458 | 111.5545 | 117.8785 | 128.4081 | 103.3614 | 119.0623 |
| TDN (μg/L) | 85.6978 | 83.8598 | 80.4642 | 78.3146 | 90.0280 | 83.4548 | 77.0374 | 72.2710 | 84.6075 | 84.3271 | 55.0125 | 70.9626 |
| PN (μg/L) | 14.9533 | 26.3551 | 21.5576 | 24.7664 | 39.6262 | 30.2492 | 21.3084 | 39.2835 | 33.2710 | 44.0810 | 48.3489 | 48.0997 |
| PIN (μg/L) | 12.4452 | 9.1736 | 7.0798 | 5.1168 | 12.0526 | 13.3612 | 9.5662 | 18.0723 | 15.1933 | 9.9588 | 20.9514 | 12.9686 |
| PON (μg/L) | 2.5081 | 17.1816 | 14.4779 | 19.6496 | 27.5736 | 16.8880 | 11.7422 | 21.2111 | 18.0777 | 34.1222 | 27.3976 | 35.1310 |
| TP (μg/L) | 32.2353 | 29.4902 | 31.6471 | 37.8235 | 38.1176 | 31.0588 | 31.9412 | 26.5490 | 36.8431 | 34.3922 | 34.6863 | 33.1176 |
| TDP (μg/L) | 21.7451 | 19.8824 | 10.3725 | 27.5294 | 23.6078 | 23.4118 | 23.1176 | 20.4706 | 24.0980 | 20.7647 | 19.6863 | 20.4706 |
| PP (μg/L) | 10.4902 | 9.6078 | 21.2745 | 10.2941 | 14.5098 | 7.6471 | 8.8235 | 6.0784 | 12.7451 | 13.6275 | 15.0000 | 12.6471 |
| POP (μg/L) | 1.3316 | 1.2718 | 3.8035 | 2.0293 | 2.2293 | 1.4911 | 1.0924 | 1.0924 | 2.0293 | 1.6107 | 2.1490 | 1.7503 |
| PIP (μg/L) | 9.1586 | 8.3360 | 17.4710 | 8.2648 | 12.2805 | 6.1559 | 7.7311 | 4.9860 | 10.7158 | 12.0167 | 12.8510 | 10.8968 |

“/” represents that the data was not measured.
